# A Scoping Review on the Attributes of Cluster Randomized Controlled Trials in Long-Term Care Facilities

**DOI:** 10.1155/2020/6085368

**Published:** 2020-01-22

**Authors:** Roni Y. Kraut, Lauren S. Katz, Oksana Babenko, Fabiola Diaz Carvallo, Roberto Alexanders, Derek S. Chan, Sandy Campbell, Dean T. Eurich, Scott Garrison

**Affiliations:** ^1^Department of Family Medicine, University of Alberta, Edmonton, Alberta, Canada T6G 2T4; ^2^Winnipeg Public Library, Winnipeg, Manitoba, Canada R3C 3P5; ^3^John W. Scott Health Sciences, University of Alberta, Edmonton, Alberta, Canada T6G 2R7; ^4^School of Public Health, University of Alberta, Edmonton, Alberta, Canada T6G 2G3

## Abstract

Cluster randomized trial design, where groups of participants are randomized instead of individual participants, is increasingly being used in long-term care research. The purpose of this review was to determine the characteristics of cluster randomized trials in long-term care facilities. A medical librarian conducted the literature search. Two independent reviewers reviewed each paper. Studies were included if the design was cluster randomized and participants were from long-term care facilities. For each included study, two independent data extractors captured data on study attributes, including: journal, location, year published, author discipline, funding, methodology, number of participants, and intervention target. The literature search yielded 7,679 unique studies, with 195 studies meeting the selection criteria and being included for data extraction. The included studies were published between 1976 and 2017, with 53% of studies published after 2009. The term cluster randomized was in the title of only 45% of the studies. The studies were conducted worldwide; the United States had the largest number of studies (23%), followed by the United Kingdom (18%). Ten percent of studies were published in journals with an impact factor >10. The most frequent discipline of the first and last authors was medicine (34%), followed by nursing (17%). Forty-nine percent of the studies had government funding, while only 20% had medical industry funding. In studies with <1000 residents, 85% of the studies obtained consent from the resident and/or their proxy, while in studies with ≥ 1000 residents, it was 31%. The most frequent intervention targets were infection (13%), falls/fracture (13%), and behavior/physical restraint (13%). Cluster randomized controlled trials in long-term care have a unique set of characteristics. Results of this review will provide guidance to researchers conducting studies in long-term care facilities.

## 1. Introduction

Long-term care facilities fulfill an important need in society. Across Europe and North America over 3 million people resided in long-term care in 2016, comprising on average 3% of adults over 65 years old [1]. Research is increasingly being conducted in this segment of the population in an effort to optimize care [2]. Cluster randomized trial is a type of randomized controlled trial, where the unit of randomization is the long-term care ward or facility instead of the individual, and is often employed in long-term care research. This design is especially well suited for evaluating group interventions in long-term care, for instance, staff education interventions or new protocols.

Knowledge of the attributes of published cluster randomized trials is advantageous for the design of future research studies. To date, there have been three systematic reviews of randomized controlled trials in long-term care, one performed by Gordon and colleagues in 2011 [2] and two performed by Diaz-Ordez and colleagues in 2013 [3, 4]. These systematic reviews focused on the type and target of interventions, consent process, and study quality [2–4].The present review builds on the previous reviews, with further characterization of cluster randomized trials. The objective of this scoping review was to determine key attributes of cluster randomized trials in long-term care.

## 2. Methods

The Preferred Reporting Items for Systematic Review and Meta-analysis (PRISMA) guidelines were used [5]. In addition, the methods followed in this review were similar to the methods in Kraut et al. 2017 systematic review [6].

### 2.1. Search Strategy

A medical librarian (S.C.) completed the database search. Databases were searched from their inception to April 1, 2017, using subject headings and text words to retrieve articles related to the concepts: “long-term care” or “nursing home” and “cluster randomization”. No limits were applied.

The search strategies were adjusted appropriately for each database. The databases in the search were: PROSPERO, Ovid MEDLINE, OVID EMBASE, Ovid Psycinfo, OVID all EBM Review databases, including the Cochrane Database of Systematic Reviews, Proquest Dissertations and Theses, and EBSCO CINAHL. Search strategies are provided in the supplementary material file. Search results were exported to RefWorks bibliographic software and duplicates were removed.

Papers that were published in languages other than English were translated using Google translate. The references of the three systematic reviews [2–4], references of the included studies from the literature search, and references of the included studies from the first reference review were checked. Authors were not contacted for additional information.

### 2.2. Study Selection

Two criteria were used to determine eligible studies. First studies had to be cluster randomized, and second studies had to be conducted in a long-term care facility. In instances where the type of facility was unclear, the following international definition of a nursing home was used: a facility that provides 24-hour functional support for people who require assistance with their activities of daily living (e.g., dressing, bathing, and eating) [7]. In instances of more than one study pertaining to the same research project, only the original study was selected.

Study selection was performed by two independent reviewers (R. Y. K., F. D. C., D. S. C., or R. A.). Any differences between reviewers were resolved through consensus, and the percentage of agreement between the reviewers was calculated.

### 2.3. Data Extraction

The following data were extracted from all included studies: (1) year published, (2) journal title, (3) study location (in cases when the study did not provide the location, the location of the authors was used as the study location), (4) names of the first and the last authors (the first and the last authors were selected as these authors typically contribute most substantially to a study), (5) highest education level of the first and the last authors (PhD, Master's, MD, undergraduate); if an author had two degrees, the higher degree was selected (for instance, for authors with both a PhD and MD, PhD was selected), (6) discipline of each author (dentistry, epidemiology , medicine, nursing, nutrition, occupational therapy, other, pharmacy, psychology, physiotherapy, social work, statistics, or unknown), (7) funder (government, foundation/charity, medical industry, or other), (8) methodology: stratified randomization, special design (stepped wedge, cross-over, factorial, or >2 groups), number of residents in a study (originally allocated after randomization), and number of clusters, (9) consent (resident and/or their proxy, only health care worker, only administration and/or indicated resident consent not required, or not stated), (10) target of intervention (behavior/physical restraint, depression, falls/fracture, infection, global function, nutrition, oral health, other, pain management, physical function/activities of daily living (ADL), prescribing, quality of life, quality of care, and skin health). When a study had fewer than four intervention targets, each target was captured. When a study had four or more intervention targets, the target was considered global function.

Author's highest education level and discipline were obtained through information in the study and Google search. Journal impact factor was obtained from the 2017 impact factor listed on the journal website. The 2017 impact factor for all the studies in this review was used due to the inflation of impact factors over time [8].

Data extraction was completed by two independent reviewers (R. Y. K., L. S. K., or F. D. C.) and differences between reviewers were resolved through consensus. The percent agreement between the reviewers was calculated.

## 3. Results

### 3.1. Study Selection

**Figure 1:**
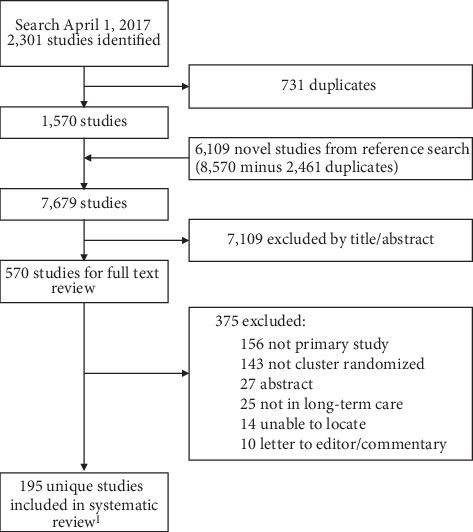
PRISMA flow diagram. ^1^195 Studies = 142 studies (from original review) + 53 studies (review of citations).

**Figure 2:**
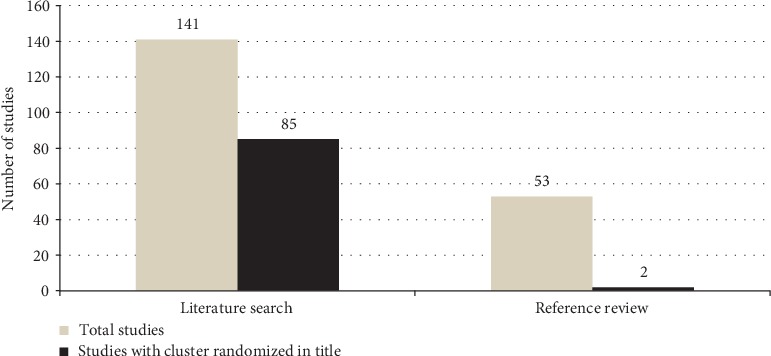
Number of studies with the term cluster randomized in their title from the literature search and the reference review.

The search results and the selection process are shown in Figure 1. There were 7,679 identified studies, and of these 195 met the selection criteria. The term cluster randomized was in the title in 60% (*n* = 85) of studies from the original search and in 2% (*n* = 2) of studies from the reference review (Figure 2). The mean agreement among the two reviewers was 87%.

### 3.2. Data Extraction

The mean agreement for data extraction between the two reviewers was 83%. The supplementary material file provides some of the characteristics of the included studies.

**Figure 3:**
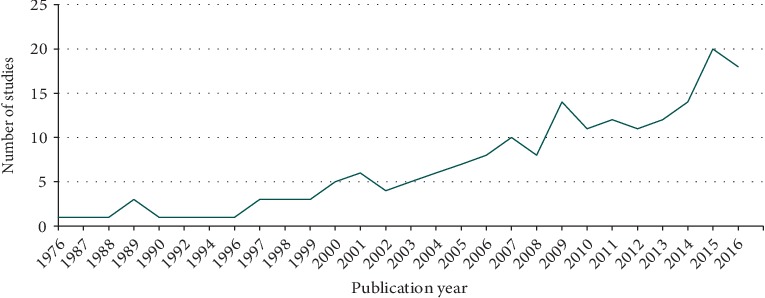
Number of published cluster randomized studies in long-term care by year.

The studies were conducted worldwide with 52% (*n* = 102) in Europe, 29% (*n* = 57) in North America, 11% (*n* = 22) in Australia/New Zealand, and 7% (*n* = 14) in Asia. The publication date of the selected studies ranged from 1976 to 2017, with 53% of the studies published after 2009 (Figure 3). The countries with over 10% of studies were the United States (23%, *n* = 45) and the United Kingdom (18%, *n* = 36).

The studies were published in 76 unique journals. The most frequent journals were the Journal of the American Geriatrics Society (12%, *n* = 28), International Journal of Geriatric Psychiatry (6%, *n* = 13), Journal of the American Medical Director's Association (5%, *n* = 12), and Age and Ageing (4%, *n* = 10). The median impact factor of the journals was 3.67 (interquartile range (IQR) = 2.78–5.06); 12% (*n* = 24) of studies were published in journals with an impact factor >10, including BMJ (*n* = 8), JAMA (*n* = 6), Lancet (*n* = 5), Archives of Internal Medicine (*n* = 2), New England Journal of Medicine (*n* = 1), Annals of Internal Medicine (*n* = 1), and the American Journal of Psychiatry (*n* = 1).

The highest education level of the first and the last authors was a PhD (55%, *n* = 215), followed by MD (16%, *n* = 61), Master's (16%, *n* = 61), undergraduate (2%, *n* = 8), other degree (3%, *n* = 12), and unknown (8%, *n* = 32). With respect to the discipline, 34% (*n* = 134) of the first and the last authors were in medicine and 17% (*n* = 65) of the authors were in nursing. Seventeen percent (*n* = 23) of physician authors and 65% (*n* = 42) of nurse authors had a PhD.

Fifty-five percent of studies (*n* = 107) had one funder, 19% (*n* = 36) had two funders, 10% (*n* = 20) had three funders, 8% (*n* = 16) had more than three funders, and in 8% (*n* = 16) of studies this information was not available. The 195 studies had in total 346 funders, of these 49% (*n* = 169) was government agency funding, 20% (*n* = 70) was foundation or charity funding, 11% (*n* = 40) was medical industry funding, 5% (*n* = 16) was other or unknown types of funding, and 15% of the studies (*n* = 52) did not disclose this information. The studies with industry funding were predominately focused on infection, falls, and oral health.

Eighty-two percent of studies (*n* = 159) had a standard design in which clusters were randomized into a control group and an intervention group and were followed over time. Ten percent of studies (*n* = 20) had more than two groups in their design, the majority of these studies had two intervention groups and one control group. Close to 3% (*n* = 5) had a stepped wedge design, 3% of studies (*n* = 6) had a factorial design, and close to 3% of studies (*n* = 5) had a cross-over design. Fifty-two percent of studies (*n* = 102) used stratified randomization. In studies with fewer than 10 clusters, 27% (*n* = 13) used stratified randomization, while in studies with 10 or more clusters, 62% (*n* = 87) used stratified randomization. The median number of participants (i.e., residents) per study was 334 (IQR = 140–742) and the median number of clusters was 15 (IQR = 9–30). The median number of clusters by the number of study participants is shown in Table 1.

Seventy-two percent of studies (*n* = 141) obtained consent from the resident and/or their proxies, 2% (*n* = 3) obtained partial consent from the resident and/or their proxies, 3% (*n* = 6) obtained consent only from health care workers, 8% (*n* = 16) obtained consent only from administration and/or the study indicated resident consent was not required, and 15% (*n* = 29) did not provide information on consent. In studies with fewer than 1000 residents, 85% obtained consent from the resident and/or their proxy, whereas in studies with 1000 or more residents, 31% obtained consent from the resident and/or their proxy (Table 1).

**Figure 4:**
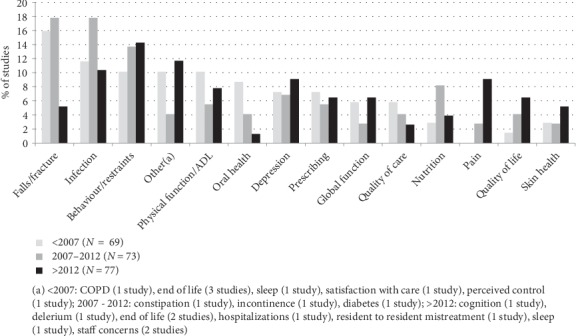
Intervention targets by year of publication.

Thirteen percent of studies (*n* = 29) focused on infection, 13% (*n* = 28) focused on falls/fractures, and 13% (*n* = 28) were on behavior/physical restraint. Other intervention targets with more than 6 studies were: physical function/activities of daily living (8%, *n* = 17), depression (8%, *n* = 17), prescribing (6%, *n* = 14), global function (5%, *n* = 11), nutrition (5%, *n* = 11), oral health (5%, *n* = 10), quality of life (4%, *n* = 9), quality of care (4%, *n* = 9), pain management (4%, *n* = 9), and skin health (4%, *n* = 9). The focus of studies changed over time, with the numbers of studies focusing on falls decreasing and studies focusing on pain increasing (Figure 4).

## 4. Discussion

Our scoping review provides a detailed characterization of published cluster randomized controlled studies in long-term care. The location of the studies in this review is consistent with the geographic distribution of the medical research publications overall, with approximately a third of the studies conducted in the US and a third in Europe [9]. In contrast, the predominant funder of the studies and the discipline of authors in this review are different from those in medical research overall [9]: only a minority of the studies in the present review were funded by medical industry and nursing was the second most frequent discipline of the authors.

The majority of studies obtained consent from the resident and/or their proxy. This is notable as consent is more challenging and resource intensive in long-term care than in the general population due to lack of competency of many long-term care residents [10].

The majority of the cluster randomized studies focused on geriatric syndromes (i.e., conditions in older people caused by a multitude of risk factors but result in the same outcome, [11]) and covered a wide range of these syndromes. Falls/fracture, infection, and behavior/restraints were studied more frequently than other syndromes, perhaps because these concerns are more pressing in long-term care facilities.

The main limitation of this study is that we may have missed some published cluster randomized trials in long-term care. We only reviewed the references of the first and second set of studies included in this review. Further our literature search terms were “long-term care” and “nursing home”, which may have missed studies in other types of long-term care facilities, for instance, geriatric hospitals. However, our search is the most rigorous of the three published systematic reviews. Diaz-Ordaz and colleagues' literature search up to early 2010 yielded 84 studies, while Gordon and colleagues' literature search up to mid 2009 yielded 77 studies [2–4].In contrast, our literature search up to the end of 2009 found 92 studies. Further, given the large number of studies in the present review, it is unlikely the missed cluster randomized trials, if any, would have a significant impact on the results.

## 5. Conclusions

Cluster randomized controlled trials in long-term care are central for advancing quality of care and translating the findings into practice. They have a unique set of characteristics, including funding, author discipline, research design, and focus. This review provides the most comprehensive review of study characteristics to further research endeavors in this field.

## Figures and Tables

**Table 1:** Number of clusters and consent by study size.

Number of participants	Number of studies	Median number of clusters (IQR)	% of studies reporting participant and/or their proxy consent
<150	49	6 (3–12)	84%
150–350	48	15 (8–21)	96%
351–999	54	14(12–21)	78%
≥1000	36	38 (20–58)	31%

IQR—interquartile range.

## Data Availability

Data and material are available from the principal investigator, who is also the corresponding author of this manuscript.

## Supplementary Materials

(Supplementary 1 Includes the literature search and the table with the characteristics of the included studies.
