# Supplementary material for: A Scoping Review on the Attributes of Cluster Randomized Controlled Trials in Long-Term Care Facilities
Source: Curr Gerontol Geriatr Res. 2020 Jan 22;2020:6085368. doi: 10.1155/2020/6085368 (PMC7204357; doi:10.1155/2020/6085368)
Supplement: (Supplementary 1 — Includes the literature search and the table with the characteristics of the included studies. [file 6085368.f1.docx]

**Literature search**

**Medline March 22, 2017**

1. ((cluster* adj2 (random* or RCT or group* or unit* or trial* or study or studies)) or (group* adj2 randomi*) or randomi?ation unit*).ti,ab.

2. exp Housing for the Elderly/ or exp Homes for the Aged/

3. exp Nursing Homes/ or exp Rehabilitation Centers/ or exp Skilled Nursing Facilities/ or (nursing home* or extended care* or care home*).mp. or ((senior* or continuing care or disabled or old age or geriatric* or elder care* or rehabilitat* or long term care) adj2 (lodge* or facility* or home* or residence* or centre* or center*)).mp.

4. (exp Nursing Homes/ or exp Residential Facilities/ or exp Rehabilitation Centers/ or exp Skilled Nursing Facilities/) and (elders or older person* or "aged, 80 and over" or older people or golden age* or elderly or geriatric* or old age or (seniors not "high school") or older adult* or centenarian* or nonagenarian* or octogenarian* or septuagenarian* or sexagenarian* or "oldest old").ti,ab.

5. (exp "Aged, 80 and over"/ or exp Geriatrics/ or exp aged/ or Health Services for the Aged/ or Senior Centers/) and (exp Nursing Homes/ or exp Residential Facilities/ or exp Rehabilitation Centers/ or exp Skilled Nursing Facilities/ or (home* or manor or manors or lodge or lodges or facility or facilities or long term care or assisted living or group homes* or "homes of aged" or nursing home* or care home* or nursing home* or extended care*)).ti,ab.

6. ((elders or older person* or "aged, 80 and over" or older people or golden age* or elderly or geriatric* or old age or (seniors not "high school") or older adult* or centenarian* or nonagenarian* or octogenarian* or septuagenarian* or sexagenarian* or "oldest old") adj3 (home* or manor or manors or lodge or lodges or facility or facilities or long term care or assisted living or group homes* or "homes for aged" or nursing home* or nursing home* or extended care* or care home*)).mp.

7. 2 or 3 or 4 or 5

8. 1 and 7

9. waiv*.mp. [mp=title, abstract, original title, name of substance word, subject heading word, floating sub-heading word, keyword heading word, protocol supplementary concept word, rare disease supplementary concept word, unique identifier, synonyms]

10. 7 and 9

11. 8 or 10

12. remove duplicates from 11

**Database: Embase <1974 to 2017 March 28>**
Search Strategy:
--------------------------------------------------------------------------------
1     ((cluster* adj2 (random* or RCT or group* or unit* or trial* or study or studies)) or (group* adj2 randomi*) or
randomi?ation unit*).ti,ab. (33132)
2     senior centre/ or elderly care/ (38307)
3     (exp nursing homes/ or residential home/) and (elders or older person* or "aged, 80 and over" or older people or
golden age* or elderly or geriatric* or old age or (seniors not "high school") or older adult* or centenarian* or
nonagenarian* or octogenarian* or septuagenarian* or sexagenarian* or "oldest old").ti,ab. (14691)
4     (exp geriatrics/ or exp aged/ or Health Services for the Aged/ or senior center/) and (exp nursing home/ or exp
residential home/ or (home* or manor or manors or lodge or lodges or facility or facilities or long term care or
assisted living or group homes* or "homes of aged" or nursing home* or care home* or nursing home* or extended
care*)).ti,ab. (106780)
5     ((elders or older person* or "aged, 80 and over" or older people or golden age* or elderly or geriatric* or old
age or (seniors not "high school") or older adult* or centenarian* or nonagenarian* or octogenarian* or septuagenarian*
or sexagenarian* or "oldest old") adj3 (home* or manor or manors or lodge or lodges or facility or facilities or long
term care or assisted living or group homes* or "homes for aged" or nursing home* or extended care* or care home*)).mp.
(9809)
6     2 or 3 or 4 or 5 (140319)
7     1 and 6 (934)
8     waiv*.mp. [mp=title, abstract, heading word, drug trade name, original title, device manufacturer, drug
manufacturer, device trade name, keyword, floating subheading] (4975)
9     6 and 8 (148)
10     7 or 9 (1079)
11     remove duplicates from 10 (1017)

**Database: PsycINFO <1806 to March Week 3 2017>**
Search Strategy:
--------------------------------------------------------------------------------
1     ((cluster* adj2 (random* or RCT or group* or unit* or trial* or study or studies)) or (group* adj2 randomi*) or
randomi?ation unit*).ti,ab. (5674)
2     (exp Nursing Homes/ or exp Long Term Care/ or exp Residential Care Institutions/) and (elders or older person* or
"aged, 80 and over" or older people or golden age* or elderly or geriatric* or old age or (seniors not "high school") or
older adult* or centenarian* or nonagenarian* or octogenarian* or septuagenarian* or sexagenarian* or "oldest
old").ti,ab. (6316)
3     exp Geriatrics/ or exp aging/ or exp elder care/ or exp Geriatric Patients/ or exp Gerontology/ (75253)
4     exp Nursing Homes/ or exp Long Term Care/ or (home* or manor or manors or lodge or lodges or facility or
facilities or long term care or assisted living or group homes* or "homes of aged" or nursing home* or care home* or
nursing home* or extended care*).ti,ab. (166979)
5     3 and 4 (10325)
6     ((elders or older person* or "aged, 80 and over" or older people or golden age* or elderly or geriatric* or old
age or (seniors not "high school") or older adult* or centenarian* or nonagenarian* or octogenarian* or septuagenarian*
or sexagenarian* or "oldest old") adj3 (home* or manor or manors or lodge or lodges or facility or facilities or long
term care or assisted living or group homes* or "homes for aged" or nursing home* or nursing home* or extended care* or
care home*)).mp. (4283)
7     2 or 5 or 6 (15170)
8     1 and 7 (89)
9     waiv*.mp. [mp=title, abstract, heading word, table of contents, key concepts, original title, tests & measures]
(1066)
10     7 and 9 (25)
11     8 or 10 (113)
12     remove duplicates from 11 (113)

**CINAHL Searched March 29, 2017**


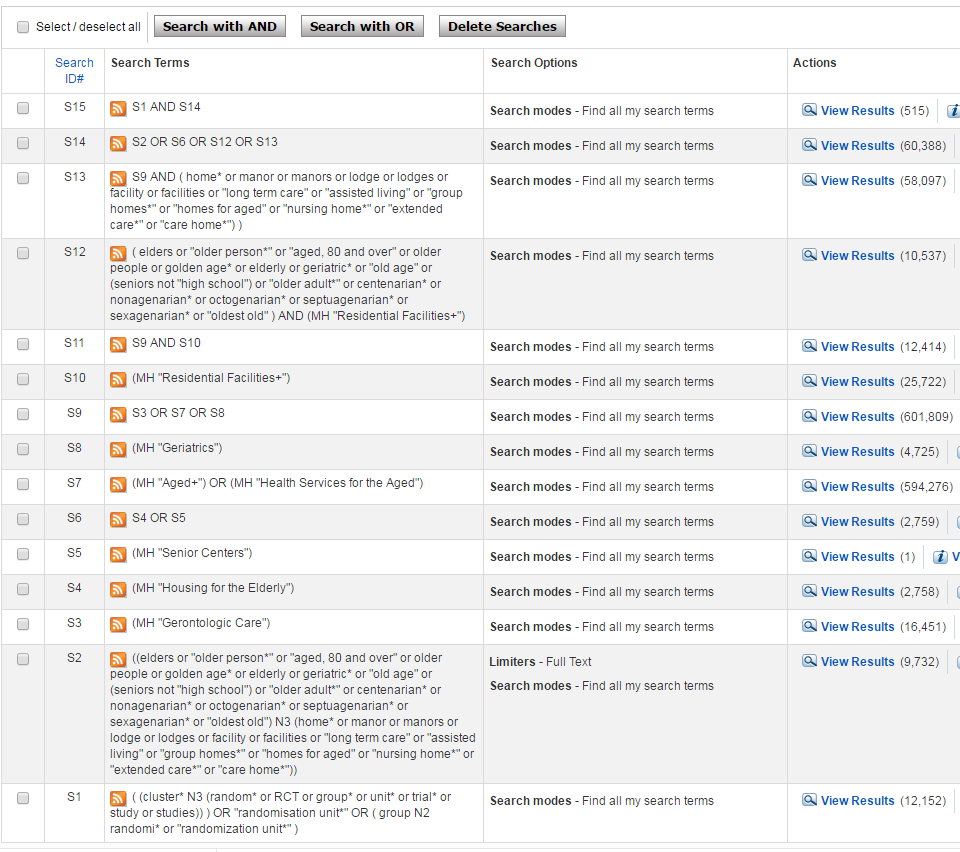


**PROSPERO**  March 29, 2017

("cluster RCT" OR "cluster randomisation" OR "cluster randomization" OR "cluster trial" OR "cluster randomized" OR "cluster randomised" OR "cluster trials") AND ("seniors center*" OR "nursing home*" OR "elder care" OR "residential facilit*") 41 studies found

**Proquest** Searched March 29, 1017 Results =21

(noft("nursing home*") OR noft("care home") OR noft("senior* center*") OR noft(residential) OR noft("long term care")) AND (noft("cluster RCT") OR noft("cluster randomisation") OR noft("cluster randomization") OR noft("cluster trial") OR noft("cluster randomized") OR noft("cluster randomised") OR noft("cluster trials")) Results =21

**Clinicaltrials.gov** Searched March 29, 2017 Results = 29

("nursing home" OR "seniors center" OR residential OR "long term care") AND ("cluster RCT" OR "cluster randomisation" OR "cluster randomization" OR "cluster trial" OR "cluster randomized" OR "cluster randomised" OR "cluster trials") | Older Adult

**Characteristics of included studies**

| **ID** | **Location** | **Stratified** | **Participant/proxy consent** | **Intervention Target** | **Number of residents** |
| --- | --- | --- | --- | --- | --- |
| Avorn 1992^1^ | United States | Yes | Not given | Prescribing - psychoactive use | 823 |
| Baldwin 2010^2^ | Northern Ireland | Yes | Yes | Infection - MRSA | 793 |
| Ballard 2016^3^ | England | Yes | Yes | Prescribing - psychoactive use, behaviour, depression | 277 |
| Barrick 2010^4^ | United States | No | Yes | Behaviour | 66 |
| Beck 2016^5^ | Denmark | No | Yes | Global function | 246 |
| Becker 2003^6^ | Germany | No | Yes | Falls - incidence | 981 |
| Beer 2011^7^ | Australia | No | Yes | Quality of life | 351 |
| Bellini 2015^8^ | Switzerland | No | Yes | Infection - MRSA | 4750 |
| Bentzen 2008^9^ | Norway | Yes | Partial | Falls - hip protector use | 1236 |
| Boorsma 2011^10^ | Netherlands | Yes | Yes | Quality of care | 462 |
| Booy 2012^11^ | Australia | Yes | Yes | Infection - Influenza | 393 |
| Bouwen 2008^12^ | Belgium | No | Yes | Falls - incidence | 379 |
| BrÃne 1989^13^ | Sweden | No | Yes | Global function | 46 |
| Bravo 2005^14^ | Canada | Yes | Yes | Quality of care | 122 |
| Brittle 2009^15^ | England | No | Yes | Physical function, depression | 56 |
| Budtz-Jorgensen 2000^16^ | Switzerland | Yes | Yes | Oral health | 237 |
| Camerson 2011^17^ | Australia | No | Yes | Falls - hip protector use | 235 |
| Carman 2000^18^ | England | Yes | Yes | Infection - Influenza | 1437 |
| Carville 2014^19^ | Australia | Yes | Yes | Skin health - skin tears | 984 |
| Chami 2012^20^ | France | Yes | No | Infection - rates | 4345 |
| Chen 2010^21^ | China (Hong Kong) | Yes | Not given | Infection - Influenza | not given |
| Chen 2015^22^ | Taiwan | No | Not given | Physical function | 127 |
| Chen 2016^23^ | Taiwan | No | Yes | Pain management | 195 |
| Cheng 2014^24^ | China (Hong Kong) | No | Yes | Cognition | 117 |
| Chenoweth 2009^25^ | Australia | Yes | Yes | Behaviour | 289 |
| Chenoweth 2014^26^ | Australia | Yes | Yes | Global function | 601 |
| Clare 2013^27^ | England | Yes | Yes | Quality of life | 65 |
| Colon-Emeric 2007^28^ | United States | No | Not given | Fractures | 606 |
| Colon-Emeric 2013^29^ | United States | Yes | No (health care worker) | Falls - incidence | 601 |
| Connolly 2015^30^ | New Zealand | Yes | No | Hospitalizations | 1998 |
| Corcoran 2017^31^ | United States | No | Yes | Physical function, nutrition | 121 |
| Cox 2008^32^ | England | Yes | Not given | Fractures | 6231 |
| Crotty 2004^33^ | Australia | No | Yes | Prescribing - appropriate | 158 |
| Davison 2007^34^ | Australia | No | Yes | Behaviour | 113 |
| Davison 2012^35^ | Australia | Yes | Yes | Depression | 216 |
| De Visschere 2011^36^ | Belgium | Yes | Yes | Oral health | 1393 |
| Drager 2016^37^ | Germany | No | Yes | Pain management | 747 |
| Drinka, 1998^38^ | United States | Yes | Yes | Infection - Influenza | 381 |
| Dyer 2004^39^ | England | No | Yes | Falls - incidence | 196 |
| Edberg 1999^40^ | Sweden | No | Not given | Behaviour, depression | 22 |
| Eisses 2005^41^ | Netherlands | Yes | Yes | Depression | 426 |
| Ersek 2016^42^ | United States | Yes | Yes | Pain management | 485 |
| Evans 1997^43^ | United States | No | Yes | Physical restraint | 463 |
| Field 2009^44^ | Canada | Yes | Not given | Prescribing - appropriate | 833 |
| Finnema 2005^45^ | Netherlands | Yes | Yes | Behaviour, depression | 194 |
| Fleet 2014^46^ | England | Yes | Yes | Infection - antibiotic prescribing | 1610 |
| Fossey 2006^47^ | England | Yes | Yes | Prescribing - psychoactive use | 349 |
| Frenkel 2001^48^ | England | No | Yes | Oral health | 378 |
| Fritsch 2009^49^ | United States | Yes | Yes | Quality of care | not given |
| Galik 2014^50^ | United states | Yes | Yes | Global function | 96 |
| Gaskill 2009^51^ | Australia | No | Yes | Nutrition | 279 |
| Gillis 2016^52^ | Belgium | No | Yes | Skin health - bathing | 163 |
| Gopal Rao 2009^53^ | England | Yes | Not given | Infection - control | 565 |
| Gudex 2010^54^ | Denmark | Yes | Yes | Global function | 348 |
| Gurwitz 2008^55^ | Canada and US | Yes | Not given | Prescribing - adverse events | 1118 |
| Hanson 2005^56^ | United states | No | No | End of life | 458 |
| Hanson 2011^57^ | United States | Yes | Yes | Nutrition | 256 |
| Hanson 2017^58^ | United States | Yes | Yes | End of life | 302 |
| Hickman 2007^59^ | United States | No | Yes | Depression | 66 |
| Ho 2012^60^ | China (Hong Kong) | No | No | Infection - rates | 2407 |
| Hoeffer 2006^61^ | United States | No | Yes | Skin health - bathing | 69 |
| Houser 2014^62^ | United States | No | Yes | Depression, behaviour | 20 |
| Hsu 2015^63^ | England | Yes | Yes | Global function | 17 |
| Huizing 2006^64^ | Netherlands | No | Yes | Physical restraint | 145 |
| Huizing 2009^65^ | Netherlands | No | Yes | Physical restraint | 371 |
| Husebo 2011^66^ | Norway | No | Yes | Behaviour | 352 |
| Jensen 2002^67^ | Sweden | Yes | Yes | Falls - incidence | 402 |
| Jeon 2015^68^ | Australia | Yes | No (health care worker) | Staff - working environment | 1730 |
| Jordan 2015^69^ | England | No | Yes | Prescribing - adverse events | 43 |
| Joronson 2015^70^ | Norway | No | Yes | Behaviour | 60 |
| Juola 2015^71^ | Finland | Yes | Yes | Prescribing - appropriate | 227 |
| Juthani-Mehta 2015^72^ | United States | Yes | Yes | Infection - pneumonia | 834 |
| Kalinowski 2015^73^ | Germany | Yes | Yes | Pain management | 737 |
| Kennedy 2015^74^ | Canada | Yes | No | Prescribing - Osteoporosis | 5478 |
| Kerse 2004^75^ | New Zealand | Yes | Yes | Falls - incidence | 553 |
| Kerse 2008^76^ | New Zealand | No | Yes | Physical function, quality of life, falls | 682 |
| Kiel 2007^77^ | United States | No | Yes | Fractures | 1042 |
| Kinley 2014^78^ | England | Yes | No | End of life | 2444 |
| Kinney 2003^79^ | United States | Yes | Not given | Activities of daily living, satisfaction with care | 2222 |
| Koczy 2011^80^ | Germany | No | No | Physical restraint | 430 |
| Koike 2009^81^ | Japan | No | Yes | Falls - incidence | 672 |
| Konner 2015^82^ | Germany | Yes | Yes | Pain management | 747 |
| Koo 2016^83^ | United States | No | Not given | Infection - control | not given |
| Kopke 2012^84^ | Germany | Yes | No | Physical restraint | 3771 |
| Kovacs 2007^85^ | Spain | No | Yes | Pain management | 673 |
| Kuck 2014^86^ | Germany | No | Yes | Sleep | 107 |
| Kuske 2009^87^ | Germany | No | Yes | Quality of care | 321 |
| Langer 1976^88^ | United States | No | Not given | Depression, behaviour, perceived control | 91 |
| Lapane 2011^89^ | United States | Yes | No | Prescribing - adverse events | 3261 |
| Law 2006^90^ | England | No | Yes | Falls - incidence | 3717 |
| Lawton 1998^91^ | United States | No | Not given | Global function | 97 |
| Lee 2002^92^ | China (hong kong) | Yes | Yes | COPD | 89 |
| Lemaitre 2009^93^ | France | Yes | Not given | Infection - Influenza | 3400 |
| Leontjevas 2013^94^ | Netherlands | No | Yes | Depression | 547 |
| Leslie 2012^95^ | England | Yes | Yes | Nutrition | 41 |
| Lin 2010^96^ | Taiwan | No | Yes | Nutrition | 85 |
| Linn 1989^97^ | United States | Yes | Yes | End of life | 306 |
| Liu 2017^98^ | China (Hong Kong) | No | Yes | Pain management | 128 |
| Loeb 2005^99^ | Canada and US | No | Not given | Infection - antibiotic prescribing | 4217 |
| Loeb 2006^100^ | Canada | Yes | Yes | Infection - pneumonia | 680 |
| Looijmans 2010^101^ | Netherlands | Yes | Not given | Infection - Influenza | 5595 |
| Lord 2003^102^ | Australia | Yes | Yes | Physical function, falls - incidence | 551 |
| Low 2013^103^ | Australia | Yes | Yes | Depression | 398 |
| MacEntee 2007^104^ | Canada | Yes | Yes | Oral health | 152 |
| MacRae 1996^105^ | United States | No | Yes | Physical function, quality of life | 37 |
| Madigan 2014^106^ | Northern Ireland | Yes | No (health care worker) | Nutrition | not given |
| Makris 2000^107^ | United States | Yes | Not given | Infection - rates | 890 |
| Mamhider 2017^108^ | Sweden | Yes | Yes | Pain management | 213 |
| McMurdo 1994^109^ | Scotland | No | Yes | Physical function | 65 |
| McMurdo 2000^110^ | Scotland | No | Not given | Falls - incidence | 133 |
| Mcsweeney 2012^111^ | Australia | No | Not given | Depression | 44 |
| Meeks 2015^112^ | United States | Yes | Yes | Depression | 82 |
| Meyer 2003^113^ | Germany | No | No | Fractures | 942 |
| Meyer 2009^114^ | Germany | No | Not given | Falls - incidence | 1125 |
| Mody 2015^115^ | United States | Yes | Yes | Infection - indwelling devices | 418 |
| Mohide 1988^116^ | Canada | Yes | Not given | Quality of care | 1525 |
| Mojon 1998^117^ | Switzerland | Yes | Yes | Oral health | 116 |
| Molloy 2000^118^ | Canada | Yes | Yes | End of life | 1292 |
| Monette 2007^119^ | Canada | Yes | No (health care worker) | Infection - antibiotic prescribing | 2168 |
| Moore 2011^120^ | Ireland | No | Yes | Skin health - pressure ulcer | 213 |
| Mozley 2007^121^ | England | No | Yes | Depression | 143 |
| Nagayama 2016^122^ | Japan | No | Yes | Quality of life, activities of daily living | 54 |
| Naughton 2001^123^ | United States | No | Not given | Infection - pneumonia | not given |
| Neyens 2009^124^ | Netherlands | Yes | Yes | Falls - incidence | 518 |
| Nijs 2006^125^ | Netherlands | Yes | Yes | Quality of life, physical function, nutrition | 178 |
| O'halloran 2004^126^ | Northern Ireland | Yes | Yes | Fractures | 4117 |
| O'Shea 2014^127^ | Ireland | Yes | Yes | Quality of life | 304 |
| Olsen 2016^128^ | Norway | No | Yes | Depression, behaviour, quality of life | 58 |
| Orrel 2007^129^ | England | No | Yes | Quality of life/unmet need | 238 |
| Patterson 2010^130^ | Northern Ireland | Yes | Yes | Prescribing - psychoactive use | 334 |
| Pellfolk 2010^131^ | Sweden | No | Not given | Physical restraint | 355 |
| Peterson 2016^132^ | United States | No | Partial | Infection - MRSA | not given |
| Pettersson 2011^133^ | Sweden | Yes | No (health care worker) | Infection - antibiotic prescribing | 2537 |
| Pieper 2016^134^ | Netherlands | No | Yes | Behaviour | 288 |
| Pitkala 2007^135^ | Finland | Yes | Yes | Constipation | 209 |
| Potter 1997^136^ | Scotland | Yes | Partial | Infection - Influenza | 1059 |
| Proctor 1999^137^ | England | Yes | Yes | Quality of care | 120 |
| Rantz 2001^138^ | United States | No | Not given | Global function | 7385 |
| Rapp 2013^139^ | Germany | No | Yes | Behaviour | 304 |
| Rasmussen 2015^140^ | Denmark | No | No (health care worker) | Staff - back pain | not given |
| Ray 1997^141^ | United States | Yes | Yes | Falls - incidence | 499 |
| Ray 2005^142^ | United States | Yes | Not given | Falls - injuries | 10558 |
| Resnick 2009^143^ | United States | Yes | Yes | Global function | 487 |
| Roberts 2001^144^ | Australia | Yes | No | Prescribing - appropriate | 3230 |
| Rockstad 2013^145^ | Norway | Yes | Yes | Behaviour | 624 |
| Rosendahl 2006^146^ | Sweden | Yes | Yes | Physical function | 191 |
| Roth 2014^147^ | Germany | No | Not given | Quality of care | 624 |
| Rothan-Tondeu 2010^148^ | France | No | No | Infection - Influenza | not given |
| Sackley 2006^149^ | England | Yes | Yes | Activities of daily living | 118 |
| Sackley 2008^150^ | England | No | Yes | Urinary continence | 34 |
| Sackley 2009^151^ | England | No | Yes | Physical function, activities of daily living | 243 |
| Sackley 2015^152^ | England | Yes | Yes | Activities of daily living | 1042 |
| Salva 2016^153^ | Spain | No | Yes | Falls - incidence | 441 |
| Sambrook 2012^154^ | Australia | No | Yes | Nutrition, falls - incidence | 602 |
| Schnelle 1999^155^ | United States | No | Yes | Sleep | 184 |
| Schoonhover 2015^156^ | Netherlands | Yes | Yes | Skin health - bathing | 500 |
| Schora 2014^157^ | United States | No | Yes | Infection - MRSA | 5828 |
| Schou 1989^158^ | Scotland | No | Yes | Oral health | 187 |
| Schrijnemaekers 2002^159^ | Netherlands | Yes | Yes | Behaviour | 151 |
| Siddiqi 2016^160^ | England | Yes | Yes | Delirium | 215 |
| Simmons 2008^161^ | United states | No | Yes | Nutrition | 124 |
| Simons 2001^162^ | England | Yes | Yes | Oral health | 164 |
| Sinclair 2012^163^ | England | No | Yes | Diabetes | 102 |
| Sloane 2004^164^ | United States | No | Yes | Skin health - bathing | 73 |
| Snyder 2013^165^ | United States | Yes | Yes | Nutrition | 256 |
| Soon 2002^166^ | Canada | Yes | Yes | Depression | 103 |
| Splett 2003^167^ | United States | No | Yes | Nutrition | 394 |
| Stein 2001^168^ | United States | Yes | Yes | Prescribing - NSAID use | 158 |
| Stern 2014^169^ | Canada | No | Yes | Skin health - pressure ulcer | 137 |
| Teresi 2013^170^ | United States | No | Yes | Resident to resident mistreatment | 1405 |
| Testad 2005^171^ | Norway | Yes | Not given | Physical restraint | 151 |
| Testad 2010^172^ | Norway | Yes | No | Behaviour | 211 |
| Testad 2015^173^ | Norway | No | Yes | Physical restraint | 274 |
| Tjla 2015^174^ | United States | Yes | No | Prescribing - psychoactive use | 5488 |
| Toots 2016^175^ | Sweden | Yes | Yes | Physical function, activities of daily living | 186 |
| Trick 2004^176^ | United States | No | Yes | Infection - MRSA, VRE | 283 |
| Tse 2012^177^ | China (Hong Kong) | No | Yes | Pain management | 535 |
| Tse 2016^178^ | China (Hong Kong) | No | Not given | Global function | 115 |
| Underwood 2013^179^ | England | Yes | Yes | Depression | 891 |
| Van de Ven 2013^180^ | Netherlands | Yes | Yes | Behaviour | 268 |
| Van der Maaden 2016^181^ | Netherlands | Yes | Yes | Infection - pneumonia | 210 |
| Van der Putten 2013^182^ | Netherlands | No | Yes | Oral health | 343 |
| Van Gaal 2011^183^ | Netherlands | Yes | Yes | Skin health - pressure ulcer, infection - UTI, falls - incidence | 392 |
| Van Malderen 2017^184^ | Belgium | No | Yes | Quality of life | 88 |
| Vigild 1990^185^ | Denmark | No | Yes | Oral health | 203 |
| Visschere 2012^186^ | Belgium | Yes | Yes | Oral health | 373 |
| Visser 2008^187^ | Australia | No | Yes | Behaviour | 76 |
| Walker 2016^188^ | England | No | Yes | Falls - incidence | 52 |
| Ward 2010^189^ | Australia | Yes | No | Fractures | 5391 |
| Wenborn 2013^190^ | England | Yes | Yes | Quality of life | 210 |
| Williams 1987^191^ | Australia | No | Not given | Global function | 20 |
| Williams 2016^192^ | United States | Yes | Yes | Behaviour | 83 |
| Yeung 2011^193^ | China (Hong Kong) | Yes | Yes | Infection - rates | 675 |
| Yokoi 2015^194^ | Japan | No | Yes | Falls - incidence | 105 |
| Zwijsen 2014^195^ | Netherlands | No | No | Behaviour | 393 |

^1^Avorn J, Soumerai S, Everitt D, Ross-Degnan D, Beers M, Sherman D, et al. A randomized trial of a program to reduce the use of

psychoactive drugs in nursing homes. N Engl J Med 1992;327:168–173.

^2^[Baldwin NS](https://www.ncbi.nlm.nih.gov/pubmed/?term=Baldwin%20NS%5BAuthor%5D&cauthor=true&cauthor_uid=20451294), [Gilpin DF](https://www.ncbi.nlm.nih.gov/pubmed/?term=Gilpin%20DF%5BAuthor%5D&cauthor=true&cauthor_uid=20451294), [Tunney MM](https://www.ncbi.nlm.nih.gov/pubmed/?term=Tunney%20MM%5BAuthor%5D&cauthor=true&cauthor_uid=20451294), [Kearney MP](https://www.ncbi.nlm.nih.gov/pubmed/?term=Kearney%20MP%5BAuthor%5D&cauthor=true&cauthor_uid=20451294), [Crymble L](https://www.ncbi.nlm.nih.gov/pubmed/?term=Crymble%20L%5BAuthor%5D&cauthor=true&cauthor_uid=20451294), [Cardwell C](https://www.ncbi.nlm.nih.gov/pubmed/?term=Cardwell%20C%5BAuthor%5D&cauthor=true&cauthor_uid=20451294), et al. Cluster randomised controlled trial of an infection control education and training intervention programme focusing on meticillin-resistant Staphylococcus aureus in nursing homes for older people. J Hosp Infect 2010;76:36-41.

^3^[Ballard C](https://www.ncbi.nlm.nih.gov/pubmed/?term=Ballard%20C%5BAuthor%5D&cauthor=true&cauthor_uid=26585409), [Orrell M](https://www.ncbi.nlm.nih.gov/pubmed/?term=Orrell%20M%5BAuthor%5D&cauthor=true&cauthor_uid=26585409), [Yong,Zhong S](https://www.ncbi.nlm.nih.gov/pubmed/?term=YongZhong%20S%5BAuthor%5D&cauthor=true&cauthor_uid=26585409), [Moniz-Cook E](https://www.ncbi.nlm.nih.gov/pubmed/?term=Moniz-Cook%20E%5BAuthor%5D&cauthor=true&cauthor_uid=26585409), [Stafford J](https://www.ncbi.nlm.nih.gov/pubmed/?term=Stafford%20J%5BAuthor%5D&cauthor=true&cauthor_uid=26585409), et al. Impact of Antipsychotic Review and Nonpharmacological Intervention on Antipsychotic Use, Neuropsychiatric Symptoms, and Mortality in People With Dementia Living in Nursing Homes: A Factorial Cluster-Randomized Controlled Trial by the Well-Being and Health for People With Dementia (WHELD) Program. [Am J Psychiatry](https://www.ncbi.nlm.nih.gov/pubmed/26585409) 2016;173:252-62.

^4^[Barrick AL](https://www.ncbi.nlm.nih.gov/pubmed/?term=Barrick%20AL%5BAuthor%5D&cauthor=true&cauthor_uid=20104513), [Sloane PD](https://www.ncbi.nlm.nih.gov/pubmed/?term=Sloane%20PD%5BAuthor%5D&cauthor=true&cauthor_uid=20104513), [Williams CS](https://www.ncbi.nlm.nih.gov/pubmed/?term=Williams%20CS%5BAuthor%5D&cauthor=true&cauthor_uid=20104513), [Mitchell CM](https://www.ncbi.nlm.nih.gov/pubmed/?term=Mitchell%20CM%5BAuthor%5D&cauthor=true&cauthor_uid=20104513), [Connell BR](https://www.ncbi.nlm.nih.gov/pubmed/?term=Connell%20BR%5BAuthor%5D&cauthor=true&cauthor_uid=20104513), [Wood W](https://www.ncbi.nlm.nih.gov/pubmed/?term=Wood%20W%5BAuthor%5D&cauthor=true&cauthor_uid=20104513), et al. Impact of ambient bright light on agitation in dementia. Int J Geriatr Psychiatry2010;25:1013-21.

^5^Beck AM, Christensen AG, Hansen BS, Damsbo-Svendsen S, Kreinfeldt Skovgaard Moller T. Multidisciplinary nutritional support for undernutrition in nursing home and home-care: A cluster randomized controlled trial. Nutrition 2016;32:199–205.

^6^Becker C, Kron M, Lindemann U, Sturm E, Eichner B, Walter-Jung B, et al. Effectiveness of a multifaceted intervention on falls in nursing home residents. J Am Geriatr Soc 2003;51:306-13.

^7^Beer C, Horner B, Flicker L, Scherer S, Lautenschlager NT, Bretland N, et al. A cluster-randomised trial of staff education to improve the quality of life

of people with dementia living in residential care: the DIRECT study. PLoS One 2011;6:e28155.

^8^Bellini C, Petignat C, Masserey E, Bula C, Burnand B, Rousson V, et al. Universal screening and decolonization for control of MRSA in nursing homes: a cluster randomized controlled study. Infect Control Hosp Epidemiol 2015;36:401–8.

^9^Bentzen H, Forsen L, Becker C, Bergland A. Uptake and adherence with soft-and hard-shelled hip protectors in Norwegian nursing homes: a cluster randomized trial. Osteoporos Int 2008;19:101-11.

^10^Boorsma M, Frijters DHM, Knol DL, Ribbe ME, Nijpels G, Van Hout HPJ. Effects of multidisciplinary integrated care on quality of care in residential care facilities for elderly people: a cluster randomized trial. CMAJ 2011;183: E724–32.

^11^Booy R, Lindley RI, Dwyer DE, Yin JK, Heron LG, Moffatt CRM, et al. Treating and preventing influenza in aged care facilities: a cluster randomised controlled trial. PLoS ONE 2012; 7:e46509.

^12^Bouwen, A, De Lepeleire, J, Buntinx, F. Rate of accidental falls in institutionalized older people with and without cognitive impairment halved as a result of a staff orientated intervention. Age and Ageing 2008;37:306–10.

^13^Brane G, Karlsson I, Kihlgren M, Norberg A: Integrity-promoting care of demented nursing home patients: psychological and biochemical changes. Int J Geriatr Psychiatry 1989;4:165–72.

^14^Bravo G, Dubois MF, Roy PM. Using Goal Attainment Scaling to improve the quality of long-term care: a group-randomized trial. Int J Qual Health Care 2005;17:511–19.

^15^Brittle N, Patel S, Wright C, Baral S, Versfeld P, Sackley C. An exploratory cluster randomized controlled trial of group exercise on mobility and depression in care home residents. Clin Rehabil 2009;23:146-54.

^16^Budtz-Jørgensen E, Mojon P, Rentsch A, Deslauriers N. Effects of an oral health program on the occurrence of oral candidosis in a long-term care facility. Community Dent Oral Epidemiol 2000;28:141-49.

^17^Cameron ID, Kurrle SE, Quine S, Sambrook PN, March L, Chan DKY, et al. Improving adherence with the use of hip protectors among older people living in nursing care facilities: a cluster randomized trial. J Am Med Dir Assoc 2011;12:50–7.

^18^Carman WF, Elder AG, Wallace LA, McAulay K, Walker A, Murray GD, et al. Effects of influenza vaccination of health-care workers on mortality of elderly people in long- term care: a randomised controlled trial. Lancet 2000;355:93–7.

^19^Carville K, Leslie G, Osseiran-Moisson R, Newall N, Lewin G. The effectiveness of a twice- daily skin-moisturizing regimen for reducing the incidence of skin tears. Int Wound J 2014;11:446–53.

^20^Chami  K, Gavazzi  G, Bar-Hen A,  Carrat F, de Wazières B, Lejeune B, et al.  A short-term, multicomponent infection control program in nursing homes: a cluster randomized controlled trial.  J Am Med Dir Assoc 2012;13:569.e9-569.e17.

^21^Chen H, Ng S, King ME., Fong C, Ng WP, Szeto KH, et al. Promotion of seasonal influenza vaccination among staff in residential care homes for elderly in Hong Kong. *Healthcare Infection* 2010;15:121-25.

^22^Chen KM, Li CH, Chang YH, Huang HT, Cheng YY. An elastic band exercise program for older adults using wheelchairs in Taiwan nursing homes: a cluster randomized trial. Int J Nurs Stud 2015;52:30-8.

^23^Chen YH, Lin LC. Ability of the pain recognition and treatment (PRT) protocol to reduce expressions of pain among institutionalized residents with dementia: a cluster randomized controlled trial. Pain Manag Nurs 2016;17:14-24

^24^Cheng ST, Chow PK, Song YQ et al: Mental and physical activities delay cognitive decline in older persons with dementia. Am J Geriatr Psychiatry 2014; 22:63‐74

^25^Chenoweth, L. King MT, Jeon YK, Brodaty H, Stein-Parbury J, Norman R,et al. Caring for aged dementia care resident study (CADRES) of person-centred care, dementia-care mapping, and usual care in dementia: a cluster-randomised trial. Lancet Neurol 2009;8:317–25.

^26^Chenoweth L, Forbes I, Fleming R, King MT, Stein-Parbury J, Luscombe G, et al. PerCEN: a cluster randomized controlled trial of person-centered residential care and environment for people with dementia. Int Psychogeriatr 2014;26:1147-60.

^27^Clare L, Whitaker R, Woods RT, Quinn C, Jelley H, Hoare Z, et al. AwareCare: a pilot randomized controlled trial of an awareness-based staff training intervention to improve quality of life for residents with severe dementia in long-term care settings. International Psychogeriatrics 2013;25: 128–39.

^28^Colon-Emeric CS, Lyles KW, House P, Levine DA, Schenck AP, Allison J, et al. Randomized trial to improve fracture prevention in nursing home residents. Am J Med 2007;120:886-92.

^29^Colon-Emeric, C.S., McConnell, E., Pinheiro, S.O., Corazzini, K., Porter, K., Earp, K.M., et al. CONNECT for better fall prevention in nursing homes: results from a pilot intervention study. J. Am. Geriatr. Soc. 2013;61:2150–59.

^30^Connolly MJ, Boyd M, Broad JB, Kerse N, Lumley T, Whitehead N, et al. The aged residential care healthcare utilization study (ARCHUS): A multidisciplinary, cluster randomized controlled trial designed to reduce acute avoidable hospitalizations from long-term care facilities. JAMDA 2015;16:49-55.

^31^Corcoran MP, Nelson ME, Sacheck JM, Reid KF, Kirn D, Fielding RA et al. Efficacy of an Exercise and Nutritional Supplement Program on Physical Performance and Nutritional Status in Older Adults With Mobility Limitations Residing at Senior Living Facilities. J. Aging Phys. Act. 2017;25:453–63.

^32^Cox H, Puffer S, Morton V, Cooper C, Hodson J, Masud T, et al. Educating nursing home staff on fracture prevention: a cluster randomised trial. Age Ageing 2008;37:167–72.

^33^Crotty M, Halbert J, Rowett D, Giles L, Birks R, Williams H. An outreach geriatric medication advisory service in residential aged care: a randomised controlled trial of case conferencing. Age Ageing 2004;33:612-7.

^34^Davison TE, McCabe MP, Visser S, Hudgson C, Buchanan G, George K. Controlled trial of dementia training with a peer support group for aged care staff. Int J Geriatr Psychiatry 2007;22:868–73.

^35^Davison TE, Karantzas G, Mellor D, McCabe MP, Mrkic D. Staff- focused interventions to increase referrals for depression in aged care facilities: A cluster randomized controlled trial. Aging & Mental Health 2013;17:449-55.

^36^De Visschere L, de Baat C, Schols J M, Deschepper E, Vanobbergen J. Evaluation of the implementation of an ‘oral hygiene protocol’ in nursing

homes: a 5-year longitudinal study. Community Dent. Oral Epidemiol 2011;39:416–25.

^37^Drager D, Budnick A, Kuhnert R, Kalinowski S, Konner F, Kreutz R. Pain management intervention targeting nursing staff and general practitioners: Pain intensity, consequences and clinical relevance for nursing home residents. Geriatr Gerontol Int. 2016; doi: 10.1111/ggi.12924.

^38^Drinka PJ, Gravenstein S, Schilling M, Krause P, Miller BA, Shult P. Duration of antiviral prophylaxis during nursing home outbreaks of influenza A: a comparison of 2 protocols. Arch Intern Med 1998;158:2155–9.

^39^Dyer CAE, Taylor GJ, Reed M, Dyer CA, Robertson DR, Harrington R. Falls prevention in residential care homes: a randomised controlled trial. Age Ageing 2004;33:596–602.

^40^Edberg AK, Norberg A, Hallberg IR. Mood and general behavior of patients with severe dementia during one year of supervised, individualized planned care and systematic clinical supervision. Comparison with a similar control group. Aging-Clinical & Experimental Research 1999;11:395–403.

^41^Eisses AMH, Kluiter H, Jongenelis K, Pot AM, Beekman ATF, Ormel J. Care staff training in detection of depression in residential homes for the elderly. Br J Psychiatry 2005;186: 404–9.

^42^Ersek M, Neradilek MB, Herr K, Jablonski A, Polissar N, Du Pen A. Pain management algorithms for implementing best practices in nursing homes: Results of a randomized controlled trial. J Am Med Dir Assoc 2016;17:348–56.

^43^Evans LK, Strumpf NE, Allen-Taylor SL, Capezuti E, Maislin G, Jacobsen B. A clinical trial to reduce restraints in nursing homes. J Am Geriatr Soc 1997;45:675–81.

^44^Field TS, Rochon P, Lee M, Gavendo L, Baril JL, Gurwitz JH. Computerized clinical decision support during medication ordering for long-term care residents with renal insufficiency. J Am Med Inform Assoc 2009;16:480–5.

^45^Finnema E, Droes RM, Ettema T, Ooms M, Ader H, Ribbe M, et al. The effect of integrated emotion-oriented care versus usual care on elderly persons with dementia in the nursing home and on nursing assistants: a randomized clinical trial. *Int J Geriatr Psychiatry* 2005;20:330–43.

^46^Fleet E, Gopal Rao G, Patel B, Cookson B, Charlett A, Bowman C , et al. Impact of implementation of a novel antimicrobial stewardship tool on antibiotic use in nursing homes: a prospective cluster randomized control pilot study. J Antimicrob Chemother 2014;69:2265–73.

^47^Fossey J, Ballard C, Juszczak E, James I, Alder N, Jacoby R, et al. Effect of enhanced psychosocial care on antipsychotic use in nursing home residents with severe dementia: cluster randomised trial. BMJ 2006;332:756–61.

^48^Frenkel HF, Harvey I, Newcombe RG. Improving oral health in institutionalised elderly people by educating caregivers: a randomised controlled trial. Community Dent Oral Epidemiol 2001;29:289–97.

^49^Fritsch T, Kwak J,Grant S,Lang J,Montgomery RR, Basting AD. Impact of TimeSlips, a creative expression intervention program, on nursing home residents with dementia and their caregivers. Gerontologist 2009;49:117-27.

^50^Galik E, Resnick B, Hammersla M, Brightwater J. Optimizing function and physical activity among nursing home residents with dementia: Testing the impact of function-focused care. Gerontologist 2013;54:930–43.

^51^Gaskill D, Isenring E, Black LJ, Hassall S, Bauer JD. Maintaining nutrition in aged care residents with a train-the-trainer intervention and Nutrition Coordinator. J Nutr Health Aging 2009;13(10):913-17.

^52^Gillis K, Tency I, Roelant E, Laureys S, Devriendt H, Lips D. Skin hydration in nursing home residents using disposable bed baths. Geriatr Nurs. 2016;37:175-79.

^53^Rao GG, Jeanes A, Russell H, Wilson D, Atere-Roberts E, O’Sullivan D, et al. Effectiveness of short-term, enhanced, infection control support in improving compliance with infection control guidelines and practice in nursing homes: a cluster randomized trial. Epidemiol infect 2009;137:1465–71.

^54^Gudex C, Horsted C, Jensen AM, Kjer M, Sørensen J. Consequences from use of reminiscence – a randomised intervention study in ten Danish nursing homes. BMC Geriatr 2010;10:33.

^55^Gurwitz JH, Field TS, Rochon P, Judge J, Harrold LR, Bell CM, et al. Effect of computerized provider order entry with clinical decision support on

adverse drug events in the long-term care setting. J Am Geriatr Soc. 2008;56:2225-33.

^56^Hanson LC, Reynolds KS, Henderson M, Pickard CG. A quality improvement intervention to increase palliative care in nursing homes. J Palliat Med 2005;8:576–84.

^57^Hanson LC, Carey TS, Caprio AJ, Lee TJ, Ersek M, Garrett J, et al. Improving decision-making for feeding options in advanced dementia: a randomized, controlled trial. J Am Geriatr Soc 2011;59:2009–16.

^58^Hanson LC, Zimmerman S, Song, MK, Lin FC., Rosemond C, Carey TS, Mitchell SL. Effect of the goals of care intervention for advanced dementia: A randomized clinical trial. JAMA Intern Med 2017;177:24–31

^59^Hickman SE, Barrick AL, Williams CS, Zimmerman S, Connell BR, Preisser JS, et al. The effect of ambient bright light therapy on depressive symptoms in persons with dementia. J Am Geriatr Soc 2007;55:1817-24.

^60^Ho ML, Seto WH, Wong LC, Wong TY. Effectiveness of multifaceted hand hygiene interventions in long-term care facilities in Hong Kong: a cluster-randomized controlled trial. Infect Control Hosp Epidemiol 2012;33:761–67.

^61^Hoeffer B, Talerico KA, Rasin J, Mitchell CM, Stewart BJ, McKenzie D, et al. Assisting cognitively impaired nursing home residents with bathing: effects of two bathing interventions on caregiving. Gerontologist 2006;46:524–32.

^62^Houser WS, George DR, and Chinchilli VM. Impact of TimeSlips creative expression program on behavioral symptoms and psychotropic medication use in persons with dementia in long-term care: a cluster- randomized pilot study..Am J Geriatr Psychiatry 2014;22:337-40.

^63^Hsu MH, Flowerdew R, Parker M, Fachner J and Odell-Miller H. Individual music therapy for managing neuropsychiatric symptoms for people with dementia and their carers: a cluster randomised controlled feasibility study. BMC Geriatr 2005;15:84.

^64^Huizing AR, Hamers JPH, Gulpers MJM, Berger MPF. Short-term effects of an educational intervention on physical restraint use: a cluster randomized trial. BMC Geriatr 2006;6:17.

^65^Huizing AR, Hamers JPH, Gulpers MJM, Berger MPF. A cluster randomized trial of an educational intervention to reduce the use of physical restraints with psychogeriatric nursing home residents. J Am Geriatr Soc 2009;57:138–48.

^66^Husebo BS, Ballard C, Sandvik R, Nilsen OB, Aarsland D. Efficacy of treating pain to reduce behavioural disturbances in residents of nursing homes with dementia: cluster randomised clinical trial. *BMJ* 2011;343:d4065.

^67^Jensen J, Lundin-Olsson L, Nyberg L, Gustafson Y. Fall and injury prevention in older people living in residential care facilities. A cluster randomized trial. Ann Intern Med 2002; 36:733–41.

^68^Jeon Y, Simpson J, Li Z, Cunich M, Thomas T, Chenoweth L, et al. Cluster randomized controlled trial of an aged care specific leadership and management program to improve work environment, staff turnover, and care quality. J Am Med Dir Assoc 2015;16:629.e19-28.

^69^Jordan S, Gabe-Walters ME, Watkins A, Humphreys I, Newson L, Snelgrove S, et al. Nurse-Led Medicines' Monitoring for Patients with

Dementia in Care Homes: A Pragmatic Cohort Stepped Wedge Cluster Randomised Trial. PLoS ONE 2015;10:e0140203.

^70^Joranson, N, Pedersen, I, Rokstad AMM, Ihlebaek C. Effects on symptoms of agitation and depression in persons with dementia participating in robot-assisted activity: a cluster-randomized controlled trial. J Am Med Dir Assoc 2015;16:867–73.

^71^Juola AL, Bjorkman MP, Pylkkanen S, Finne-Soveri H, Soini H, Kautiainen H, et al. Feasibility and baseline findings of an educational intervention in a randomized trial to optimize drug treatment among residents in assisted living facilities. Eur Geriatr Med 2014;5:195-9.

^72^Juthani-Mehta M, Van Ness PH, McGloin J, Argraves S, Chen S, Charpentier P, et al. A cluster-randomized controlled trial of a multicomponent intervention protocol for pneumonia prevention among nursing home elders. Clin Infect Dis 2015;60:849–57.

^73^Kalinowski S, Budnick A, Kuhnert R, Konner F, Kissel-Kroll A, Kreutz R et al. Nonpharmacologic pain management interventions in German nursing homes: a cluster randomized trial. Pain Manag Nurs. 2015;16:464‐74.

^74^Kennedy CC, Ioannidis G, Thabane L, Adachi JD, Marr S, Giangregorio LM, et al. Successful knowledge translation intervention in long-term care: final results from the vitamin D and osteoporosis study (ViDOS) pilot cluster randomized controlled trial. Trials. 2015;16:214.

^75^Kerse N, Butler M, Robinson E, Todd M. Fall prevention in residential care: A cluster, randomized, controlled trial. J Am Geriatr Soc 2004;52:524–31.

^76^Kerse N, Peri K, Robinson E, Wilkinson T, von Randow M, Kiata L, et al. Does a functional activity programme improve function, quality of life, and falls for residents in long term care? Cluster randomised controlled trial. BMJ 2008;337:a1445.

^77^Kiel DP, Magaziner J, Zimmerman S, Ball L, Barton BA, Brown KM. Efficacy of a hip protector to prevent hip fracture in nursing home residents: The HIP PRO randomized controlled trial. JAMA 2007;298:413–22.

^78^Kinley J, Stone L, Dewey M, Levy J, Stewart T, McCrone P, et al. The effect of using high facilitation when implementing the Gold Standards Framework in Care Homes programme: a cluster randomised controlled trial. Palliat Med 2014; 28:1099–1109.

^79^Kinney ED, Kennedy J, Loveland Cook CA, Freedman JA, Lane KA, Hui SL. A randomized trial of two quality improvement strategies implemented in a statewide public community-based, long-term care program. Med Care 2003;41:1048–57.

^80^Koczy P, Becker C, Rapp K, Klie T, Beische D, Buchele G, et al. Effectiveness of a multifactorial intervention to reduce physical restraints in nursing home residents. J Am Geriatr Soc 2011;59:333–39.

^81^Koike T, Orito Y, Toyoda H, Tada M, Sugama R, Hoshino M, et al. External hip protectors are effective for the elderly with higher-than-average risk factors for hip fractures. Osteoporos Int 2009;1613–20.

^82^Könner F, Budnick A, Kuhnert R, Wulff I, Kalinowski S, Martus P. Interventions to address deficits of pharmacological pain management in nursing home residents—A cluster randomized trial. Eur J Pain 2015;19:1331-41.

^83^Koo E, McNamara S, Lansing B, Olmsted RN, Rye RA, Fitzgerald T et al. Making infection prevention education interactive can enhance knowledge and improve outcomes: Results from the Targeted Infection Prevention (TIP) study. Am J Infect Control 2016;44:1241–46.

^84^Kopke S, Muhlhauser I, Gerlach A, Haut A, Haastert B, Mohler R, et al. Effect of a guideline-based multi-component intervention on use of physical restraints in nursing homes: A randomized controlled trial. JAMA 2012;307:2177–84.

^85^Kovacs F, Abraira V, Santos S, Diaz E, Gestoso M, Muriel A et al. A comparison of two short education programs for improving low back pain-related disability in the elderly: A cluster randomized controlled trial. Spine 2007;32:1053–59.

^86^Kuck, J, Pantke, M, Flick, U. Effects of social activation and physical mobilization on sleep in nursing home residents. Geriatr Nurs 2004;35:455-61.

^87^Kuske B, Luck T, Hanns S, Matschinger H, Angermeyer MC, Behrens J, et al. Training in dementia care: A cluster-randomized controlled trial of a training program for nursing home staff in Germany. Int Psychogeriatr 2009;21:295–308.

^88^Langer EJ, Rodin J. The effects of choice and enhanced personal responsibilities for the aged: a field experiment in an institutional setting. J Pers Soc Psychol 1976;34:191-98.

^89^Lapane KL, Hughes CM, Daiello LA, Cameron KA, Feinberg J. Effect of a pharmacist-led multicomponent intervention focusing on the medication monitoring phase to prevent potential adverse drug events in nursing homes. J Am Geriatr Soc 2011;59:1238-45.

^90^Law M, Withers H, Morris J, Anderson F. Vitamin D supplementation and the prevention of fractures and falls: results of a randomised trial in elderly people in residential accommodation. Age Ageing 2006;35:482–86.

^91^Lawton MP, Van Haitsma K, Klapper J, Kleban MH, Katz IR, Corn J: A stimulation-retreat special care unit for elders with dementing illness. Int Psychogeriatr 1998; 10:379–95.

^92^Lee DT, Lee IF, Mackenzie AE, Ho RN. Effects of a care protocol on care outcomes in older nursing home patients with chronic obstructive pulmonary disease. J Am Geriatr Soc 2002;50: 870–6.

^93^Lemaitre M, Meret T, Rothan-Tondeur M, Belmin J, Lejonv JL, Luquel L. Effect of influenza vaccination of nursing home staff on mortality of residents: a cluster- randomized trial. J Am Geriatr Soc 2009;57:1580–6.

^94^Leontjevas R, Gerritsen DL, Smalbrugge M, Teerenstra S, Vernooij-Dassen MJ, Koopmans RT. A structural multidisciplinary approach to depression management in nursing-home residents: a multicentre, stepped-wedge cluster- randomised trial. Lancet 2013; 381:2255–64.

^95^Leslie WS, Woodward M, Lean MEJ, Theobald H, Watson L, Hankey CR. Improving the dietary intake of under nourished older people in residential care homes using an energy-enriching food approach: a cluster randomised controlled study. J Hum Nutr Diet 2013;26:387–94.

^96^Lin LC, Huang YJ, Su SG, Watson R, Tsai BWJ, Wu, SC. Using spaced retrieval and Montessori-based activities in improving eating ability for residents with dementia. Int J Geriatr Psychiatry 2010;25:953-59.

^97^Linn MW, Linn BW, Stein S, Stein EM. Effect of nursing home staff training on quality of patient survival. Int J Aging Hum Dev 1989;28:305-315.

^98^Liu JYW, Lai CKY. Implementation of observational pain management protocol for residents with dementia: a cluster-RCT. J Am Geriatr Soc 2017;65:e56-63.

^99^Loeb M, Brazil K, Lohfeld L, McGeer A, Simor A, Stevenson K. Effect of a multifaceted intervention on number of antimicrobial prescriptions for suspected urinary tract infections in residents of nursing homes: cluster randomised controlled trial. BMJ 2005;331:669.

^100^Loeb M, Carusone SC, Goeree R, Walter SD, Brazil K, Krueger P, et al. Effect of a clinical pathway to reduce hospitalizations in nursing home residents

with pneumonia: a randomized controlled trial. JAMA 2006;295:2503-10.

^101^Looijmans-van, den Akker I, van Delden JJ, Verheij TJ, van der Sande MA, van Essen GA, et al. Effects of a multi-faceted program to increase influenza vaccine uptake among health care workers in nursing homes: a cluster randomised control trial. Vaccine 2010;28:5086–92.

^102^Lord SR, Castell S, Corcoran J, Dayhew J, Matters B, Shan A, et al. The effect of group exercise on physical functioning and falls in frail older people living in retirement villages: a randomized, controlled trial. J Am Geriatr Soc 2003;51:1685–92.

^103^Low LF, Brodaty H, Goodenough B, Spitzer P, Bell JP, Fleming R, et al: The Sydney Multisite Intervention of LaughterBoss and ElderClowns (SMILE) study: cluster randomised trial of humour therapy in nursing homes. BMJ Open 2013;3.

^104^MacEntee MI, Wyatt CC, Beattie BL, Paterson B, Levy- Milne R, McCandless L, et al. Provision of mouth care in long-term care facilities: an educational trial. Community Dent Oral Epidemiol 2007;35(1):25–34.

^105^MacRae PG, Asplund LA, Schnelle JF, Ouslander JG, Abrahamse A, Morris C, 1996. A walking program for nursing home residents: effects on walk endurance, physical activity, mobility, and quality of life. J Am Geriatr Soc 1996;44;175–80.

^106^Madigan SM, Fleming P, Wright ME, Stevenson M, Macauley D. A cluster randomised controlled trial of a nutrition education intervention in the community. *J Hum Nutr Diet* 2014;27:12-20.

^107^Makris AT, Morgan L, Gaber DJ, Richter A, Rubino J. Effect of a comprehensive infection control program on the incidence of infections in long-term care facilities. Am J Infect Control 2000;28:3-7.

^108^Mamhidir AG, Sjolund BM, Flackman B, Wimo A, Skoldunger A, and Engstrom M. Systematic pain assessment in nursing homes: a cluster-randomized trial using mixed methods approach. BMC Geriatrics 2017;17:61.

^109^McMurdo MET, Rennie LM. Improvements in quadriceps strength with regular exercise in the institutionalized elderly. Arch Phys Med Rehabil 1994;75:600-3.

^110^McMurdo, MET, Millar A, Daly F. A randomized controlled trial of fall prevention strategies in old peoples’ homes. Gerontology 2000;46:83-87.

^111^McSweeney K, Jeffreys A, Griffith J, Plakiotis C, Kharsas R, O;Connor DW. Specialist mental health consultation for depression in Australian aged care residents with dementia: a cluster randomized trial. Int J Geriatr Psychiatry 2012;27:1163-71.

^112^Meeks S, Van Haitsma K, Schoenbachler B, Looney, SW. BE-ACTIV for depression in nursing homes: primary outcomes of a randomized clinical trial. J Gerontol B Psychol Sci and Soc Sci 2015;70:13–23.

^113^Meyer G, Warnke A, Bender R, Muhlhauser I*.* Effect on hip fractures of increased use of hip protectors in nursing homes: Cluster randomized controlled trial. BMJ 2003;326:76-8.

^114^Meyer G, Kopke S, Haastert B, Muhlhauser I. Comparison of a fall risk assessment tool with nurses' judgement alone: a cluster-randomised controlled trial. Age and Ageing 2009;38:417-23.

^115^Mody L, Krein SL, Saint SK, Min LC, Montoya A, Lansing B ,et al. A targeted infection prevention intervention in nursing home residents with indwelling devices: A randomized clinical trial. JAMA Intern Med 2015;175:714–23.

^116^Mohide EA, Tugwell PX, Caulfield PA, Chambers LW, Dunnett CW, Baptiste S, et al. A randomized trial of quality assurance in nursing homes. Med Care 1988;26:554-65.

^117^Mojon P, Rentsch A, Budtz-Jorgensen E, Baehni PC. Effects of an oral health program on selected clinical parameters and salivary bacteria in a long-term care facility. Eur J Oral Sci 1998;106:827–34.

^118^Molloy DW, Guyatt GH, Russo R, Goeree R, O’Brien BJ, Bedard M, et al. Systematic implementation of an advance directive program in nursing homes: a randomized controlled trial. JAMA. 2000;283:1437-44.

^119^Monette J, Miller MA, Monette M, Laurier C, Boivin JF, Sourial N, et al. Effect of an educational intervention on optimizing antibiotic prescribing in long-term care facilities. J Am Geriatr Soc 2007;55:1231-5.

^120^Moore Z, Cowman S, Conroy RM. A randomised controlled clinical trial of repositioning, using the 30° tilt, for the prevention of pressure ulcers. J Clin Nurs 2011;20:2633-44.

^121^Mozley CG, Schneider J, Cordingley L, Molineux M, Duggan S, Hart C, et al. The Care Home Activity Project: does introducing an occupational therapy programme reduce depression in care homes? Aging and Mental Health 2007;11: 99-107.

^122^Nagayama H, Tomori K, Ohno K, Takahashi K, Ogahara K, Sawada T, et al. (2016) Effectiveness and Cost-Effectiveness of Occupation-Based Occupational Therapy Using the Aid for Decision Making in Occupation Choice (ADOC) for Older Residents: Pilot Cluster Randomized Controlled Trial. PLoS ONE 11(3): e0150374.

^123^Naughton B, Mylotte J, Ramadan F, Karuza J, Priore, R. Antibiotic use, hospital admissions, and mortality before and after implementing guidelines for nursing home-acquired pneumonia. J Am Geriatr Soc 2001;49:1020–24.

^124^Neyens J, Dijcks B, Twisk J, Schols J, van Haastregt J, van den Heuvel J, et al. A multifactorial intervention for the prevention of falls in psychogeriatric nursing home patients, a randomised controlled trial (RCT). Age Ageing 2009;38:194–99.

^125^Nijs KA, Graaf de C, Kok FJ, Staveren van WA. Effect of family style mealtimes on quality of life, physical performance and body weight of nursing home residents: cluster randomised controlled trial. BMJ 2006;332:1180–3.

^126^O'Halloran PD,  CranG,  Beringer TR, et al. A cluster randomised controlled trial to evaluate a policy of making hip protectors available to residents of nursing homes. Age Ageing 2004;33:582-8.

^127^O’Shea E, Devane D, Cooney A, Casey D, Jordan F, Hunter A, et al. The impact of reminiscence on the quality of life of residents with dementia in long-stay care. Int J Geriatr Psychiatry 2004;29:1062–70.

^128^Olsen C, Pedersen I, Bergland A, Enders-Slegers M, Patil G, Ihlebaek C. Effect of animal-assisted interventions on depression, agitation and quality of life in nursing home residents suffering from cognitive impairment or dementia: A cluster randomized controlled trial. Int J Geriatr Psychiatry 2016;31:1312-21.

^129^Orrell M, Hancock G, Hoe J, Woods B, Livingston G, Challis D. A cluster randomised controlled trial to reduce the unmet needs of people with dementia living in residential care. Int J Geriatr Psychiatry 2007;22:1127–34.

^130^Patterson SM, Hughes CM, Crealey G, Cardwell C, Lapane KL. An evaluation of an adapted U.S. model of pharmaceutical care to improve psychoactive prescribing for nursing home residents in northern ireland (fleetwood northern ire- land study). J Am Geriatr Soc. 2010;58:44-53.

^131^Pellfolk TJ, Gustafson Y, Bucht G et Karlsson S. Effects of a restraint minimization program on staff knowledge, attitudes, and practice: A cluster randomized trial. J Am Geriatr Soc 2010;58:62–69.

^132^Peterson LR, Boehm S, Beaumont JL, Patel PA, Schora DM, Peterson KE. Et al. Reduction of methicillin-resistant Staphylococcus aureus infection in long-term care is possible while maintaining patient socialization: a prospective randomized clinical trial. Am J Infect Control 2016;44:1622–7.

^133^Pettersson E, Vernby A, Molstad S, Lundborg CS. Can a multifaceted educational intervention targeting both nurses and physicians change the prescribing of antibiotics to nursing home residents? A cluster randomized controlled trial. J Antimicrob Chemother 2011;66:2659-66.

^134^Pieper MJ, Francke AL, van der Steen JT, Scherder EJ, Twisk JW, Kovach CR, et al. Effects of a stepwise multidisciplinary intervention for challenging behavior in advanced dementia: A cluster randomized controlled trial. J Am Geriatr Soc 2016;64:261–9.

^135^Pitkala KH, Strandberg TE, Finne Soveri UH, Ouwehand AC, Poussa T, Salminen S. Fermented cereal with specific bifidobacteria normalizes bowel movements in elderly nursing home residents. A randomized, controlled trial. J Nutr Health Aging 2007;11:305–11.

^136^Potter J, Stott DJ, Roberts MA, Elder AG, O’Donnell B, Knight PV, et al. Influenza vaccination of health care workers in long-term-care hospitals reduces the mortality of elderly patients. J Infect Dis 1997;175:1-6.

^137^Proctor R, Burns A, Powell HS, Tarrier N, Faragher B, Richardson,G., et al. Behavioural management in nursing and residential homes: A randomised controlled trial. Lancet 1999;354:26–29.

^138^Rantz, MJ, Popejoy L, Petroski GF, Madsen RW, Mehr DR, Zwygart-Stauffacher M, et al. Randomized clinical trial of a quality improvement intervention in nursing homes. The Gerontologist 2001;41:525-38.

^139^Rapp MA, Mell T, Majic T, Treusch Y, Nordheim J, Niemann-Mirmehdi M, et al. Agitation in nursing home residents with dementia (VIDEANT trial): effects of a cluster-randomized, controlled, guideline implementation trial. J Am Med Dir Assoc. 2013;14:690–5.

^140^Rasmussen CDN, Holtermann A, Bay H, Sogaard K, Jorgensen MB. A multifaceted workplace intervention for low back pain in nurses’ aides: a pragmatic stepped wedge cluster randomised controlled trial Pain 2015;156:1786–94.

^141^Ray WA, Taylor JA, Meador KG, Thapa PB, Brown AK, Kajihara HK, et al. A randomized trial of a consultation service to reduce falls in nursing homes. JAMA 1997;278:557-62.

^142^Ray, WA, Taylor, JA, Brown, AK, Gideon, P, Hall, K, Arbogast, P et al. Prevention of fall-related injuries in long-term care: a randomized controlled trial of staff education. Arch Intern Med 2005;165:2293–98.

^143^Resnick B, Gruber-Baldini A, Zimmerman S, Galik E, Pretzer-Aboff I, Russ K, et al. Nursing home resident outcomes from the Res-Care intervention. J Am Geriatr Soc 2009;57:1156–65.

^144^Roberts M, Stokes J, King M, Lynne TA, Purdie DM, Glasziou PP, et al. Outcomes of a randomised controlled trial of a clinical pharmacy intervention in 52 nursing homes. Br J Clin Pharmacol 2001;51:257-65.

^145^Rokstad, AM, Rosvik J, Kirkevold O, Selbaek G, Saltyte Benth J, Engedal, K. The effect of person-centred dementia care to prevent agitation and other neuropsychiatric symptoms and enhance quality of life in nursing home patients: a 10-month randomized controlled trial. Dement Geriatr Cogn Disord 2013;36:340–53.

^146^Rosendahl E, Lindelof N, Littbrand H, Yifter-Lindgren E, Lundin-Olsson L, et al. 2006. High-intensity functional exercise program and protein-enriched energy supplement for older persons dependent in activities of daily living: a randomised controlled trial. Aus J Physiother 2006;52:105–13.

^147^Roth Günter, Wolter A, Stolle C, Rothgang H. The long and bumpy road to outcome oriented management of long term care in Germany: implementation of the Resident Assessment Instrument in home care services. Int J Health Plan Manage 2014;29:316-29.

^148^Rothan-Tondeur M, Filali-Zegzouti Y, Belmin J, Lejeune B, Golmard JL, de Wazières B, et al. Assessment of healthcare worker influenza vaccination program in French geriatric wards: a cluster-randomized controlled trial. Aging Clin Exp Res 2010; 22:450-5.

^149^Sackley C, Wade DT, Mant D, Copley Atkinson J, Yudkin P, Cardoso K, et al. Cluster randomized pilot controlled trial of an occupational therapy intervention for residents with stroke in UK care homes. Stroke 2006;37:2336–41.

^150^Sackley CM, Rodriguez NA, van der Berg M, Badger F, Wright CC, Besemer J, et al. A phase II exploratory cluster randomised controlled trial of a group mobility training and staff education intervention to promote urinary continence in UK care homes. Clin Rehabil 2008;22:714-21.

^151^Sackley CM, van den Berg ME, Lett K, Patal S, Hollands K, Wright C et al. Effects of a physiotherapy and occupational therapy intervention on mobility and activity in care home residents: a cluster randomised controlled trial. BMJ 2009;339:b3123.

^152^Sackley CM, Walker MF, Burton CR, Watkins CL, Mant J, Roalfe A, et al. An occupational therapy intervention for residents with stroke related disabilities in UK care homes (OTCH): cluster randomised controlled trial. BMJ 2015;350:h468.

^153^Salvà A, Rojano X, Coll-Planas L, Domènech S, Roqué M. Ensayo clínico aleatorizado de una estrategia de prevención de caídas en ancianos institucionalizados basada en el Mini Falls Assessment Instrument. Rev Esp Geriatr Gerontol 2016;51:18–24.

^154^Sambrook PN, Cameron ID, Chen JS, Cumming RG, Durvasula S, Herrmann M, et al. Does increased sunlight exposure work as a strategy to improve vitamin D status in the elderly: a cluster randomised controlled trial. Osteoporos Int. 2012;23:615–24.

^155^Schnelle JF, Alessi CA, Al-Samarrai NR, Fricker RD, Ouslander JG. The nursing home at night: effects of an intervention on noise, light and sleep. J Am Geriatr Soc 1999;47:430-38.

^156^Schoonhoven L, van Gaal BG, Teerenstra S, Adang E, van der Vleuten C, van Achterberg T. Cost-consequence analysis of washing without water for nursing home residents: a cluster randomized trial. Int J Nurs Stud 2015;52: 112–20.

^157^Schora DM, Boehm S, Das S, Patel PA, O’Brien J, Hines C, et al. Impact of detection, education, research and decolonization without isolation in long-term care (DERAIL) on methicillin- resistant Staphylococcus aureus colonization and transmission at 3 long-term care facilities. Am J Infect Control 2014;42:S269–73.

^158^Schou L, Wight C, Clemson N, Douglas S, Clark C. Oral health promotion for institutionalised elderly. Community Dent Oral Epidemiol 1989;17:2-6.

^159^Schrijnemaekers V, van Rossum E, Candel M, Frederiks C, Derix M, Sielhorst H, et al. Effects of emotion-oriented care on elderly people with cognitive impairment and behavioral problems. Int J Geriatr Psychiatry 2002;17:926–37.

^160^Siddiqi N, Cheater D, Collinson M, Farrin A, Forster A, George D, et al. The PiTSTOP study: a feasibility cluster randomized trial of delirium prevention in care homes for older people. Age and Ageing 2016;45:652-661.

^161^Simmons SF, Keeler E, Zhuo X, Hickey KA, Sato HW, and Schnelle JF. Prevention of unintentional weight loss in nursing home residents: a controlled trial of feeding assistance. J Am Geriatr Soc. 2008;56:1466–73.

^162^Simons D, Brailsford S, Kidd EA, Beighton D. The effect of chlorhexidine/xylitol chewing gum on the plaque and gingival indices of elderly occupants in residential homes. J Clin Perio 2001;28:101–5.

^163^Sinclair AJ, Girling AJ, Gadsby R, Bourdel-Marchasson I, Bayer AJ. Diabetes in care homes: A cluster randomised controlled trial of resident education. Br J Diabetes Vasc Dis 2012;12:238-42.

^164^Sloane PD, Hoeffer B, Mitchell CM, McKenzie DA, Barrick AL, Rader J, et al. Effect of person-centered showering and the towel bath on bathing-associated Aggression, agitation, and discomfort in nursing home residents with dementia: a randomized, controlled trial. J Am Geriatr Soc 2004;52:1795-804.

^165^Snyder EA, Caprio AJ, Wessell K, Lin FC, Hanson LC. Impact of a decision aid on surrogate decision-makers’ perceptions of feeding options for patients with dementia. J Am Med Dir Assoc 2013;14:114–8.

^166^Soon, JA, Levine M. Screening for depression in patients in long-term care facilities: a randomized controlled trial of physician response. J Am Geriatr Soc 2002;,50:1092-99.

^167^Splett PL, Roth-Yousey LL, Vogelzang JL. Medical nutrition therapy for the prevention and treatment of unintentional weight loss in residential healthcare facilities. J Am Diet Assoc 2003;103:352-62.

^168^Stein C, Griffin MR, Taylor JA, Pichert, JW, Brandt KD, Ray WA, et al. Educational program for nursing home physicians and staff to reduce use of non-steroidal anti-inflammatory drugs among nursing home residents: a randomized controlled trial. Med Care 2001;39:436–45.

^169^Stern A, Mitsakakis N, Paulden M, Alibhai S, Wong J, Tomlinson G, et al. Pressure ulcer multidisciplinary teams via telemedicine: a pragmatic cluster randomized stepped wedge trial in long term care. BMC Health Serv Res. 2014;14:83.

^170^Teresi JA, Ramirez M, Ellis J, Silver S, Boratgis G, Kong J, et al. A staff intervention targeting resident-to- resident elder mistreatment (RREM) in long-term care increased staff knowledge, recognition and reporting: Results from a cluster randomized trial. Int J Nurs Stud 2013;50:644–56.

^171^Testad I, Aasland AM, Aarsland D. The effect of staff training on the use of restraint in dementia: a single-blind randomized controlled trial. Int J Geriatr Psychiatry 2005;20:587-90.

^172^Testad I, Ballard C, Brønnick K, Aarsland, D. The effect of staff training on agitation and use of restaint in nursing home residents with dementia: a single-blind, randomized control trial. Journal of Clinical Psychiatry 2010;71:80–86.

^173^Testad I, Mekki TE, Forland O, Oye C, Tveit EM, Jacobsen F. Modelling and evaluating evidence-based continuing education program in nursing home dementia care (MEDCED). Training of care home staff to reduce use of restraint in care home residents with dementia. A cluster randomized controlled trial. Int J Geriatr Psychiatry 2015;31:24–32.

^174^Tjia  J, Field  T, Mazor  K,  Lemay CA, Kanaan AO, Donovan JL,et al.  Dissemination of evidence-based antipsychotic prescribing guidelines to nursing homes: a cluster randomized trial.  J Am Geriatr Soc 2015;63:1289-98.

^175^Toots A, Littbrand H, Lindelöf N, Wiklund R, Holmberg H, Nordstr öm, et al. Effects of a high-intensity functional exercise program on dependence in activities of daily living and balance in older adults with dementia. J Am Geriatr Soc 2016;64:55–64.

^176^Trick WE, Weinstein RA, DeMarais PL, Tomaska W, Nathan C, McAllister SK, et al. Comparison of routine glove use and contact-isolation precautions to prevent transmission of multidrug-resistant bacteria in a long-term care facility. J Am Geriatr Soc 2004;52:2003–9.

^177^Tse MMY, Vong SKS & Ho SSK. The effectiveness of an integrated pain management program for older persons and staff in nursing homes. Arch Gerontol Geriatr 2012;54:e203–12.

^178^Tse M, Ng S, Lee P, Lai C, Kwong E, Liu J. Play activities program to relieve chronic pain and enhance functional mobility and psychological well-being for frail older adults: a pilot cluster randomized controlled trial. J Am Geriatr Soc; 64:e86-8.

^179^Underwood M, Lamb SE, Eldridge S, Sheehan B, Slowther AM, Spencer A, et al. Exercise for depression in elderly residents of care homes: a cluster-randomized controlled trial. Lancet 2013;382: 41–9.

^180^Van De Ven G, Draskovic I, Adang EMM, Donders R, Zuidema SU, et al. Effects of dementia-care mapping on residents and staff of care homes: a pragmatic cluster-randomised controlled trial. PLoS One 2013;8:e67325.

^181^Van der Maaden T, de Vet HCW, Achterberg WP, Boersma F, Schols JMGA, Mehr D, et al. Improving comfort in people with dementia and pneumonia: a cluster randomized trial. BMC Med 2016;14:116.

^182^van der Putten GJ, Mulder J, de Baat C, De Visschere LMJ, Vanobbergen JNO, Schols JMGA. Effectiveness of supervised implementation of an oral health care guideline in care homes; a single- blinded cluster randomized con- trolled trial. Clin Oral Invest 2013;17:1143–53.

^183^van Gaal BGI, Schoonhoven L, Mintjes JAJ, Borm GF, Hulscher MEJL, Defloor T, et al. Fewer adverse events as a result of the SAFE or SORRY? Programme in hospitals and nursing homes. Part I: primary outcome of a cluster randomised trial. Int J Nurs Stud 2011;48:1040–8.

^184^Van Malderen L, De Vriendt P, Mets T, Verte D, Gorus E. Experiences and effects of structurally involving residents in the nursing home by means of participatory action research: a mixed method study. J Am Med Dir Assoc 2017;18:495-502.

^185^Vigild M. Evaluation of an oral health service for nursing home residents, Acta Odontol Scand 1990;48:99-105.

^186^De Visschere L, Schols J, Van Der Putten GJ, de Baat C, Vanobbergen J. Effect evaluation of a supervised versus non-supervised implementation of an oral health care guideline in nursing homes: a cluster randomised controlled clinical trial. Gerodontology 2012;29: e96–106.

^187^Visser SM, McCabe MP, Hudgson C, Buchanan G, Davison TE, George K. Managing behavioural symptoms of dementia: effectiveness of staff education and peer support. Aging Ment Health 2008;12:47–55.

^188^Walker GM, Armstrong S, Gordon AL, Gladman J, Robertson K, Ward M, et al. The falls in care home study: a feasibility randomized controlled trial of the use of a risk assessment and decision support tool to prevent falls in care homes. Clin Rehabil 2016;30:972–83.

^189^Ward JA, Harden M, Gibson RE, Byles JE. A cluster randomised controlled trial to prevent injury due to falls in a residential aged care population, Med J Aust 2010;192:319–322.

^190^Wenborn J, Challis D, Head J, Miranda-Castillo C, Popham C, Thakur R, et al. Providing activity for people with dementia in care homes: a cluster randomised controlled trial. Int J Geriatr Psychiatry 2013:28:1296–304.

^191^Williams R, Reeve W, Ivison D, Kavanagh D. Use of environmental manipulation and modified informal reality orientation with institutionalized confused elderly subjects: a replication. Age and Ageing 1987;16:315-18.

^192^Williams KN, Perkhounkova Y, Herman R, Bossen A. A Communication Intervention to Reduce Resistiveness in Dementia Care: A Cluster Randomized Controlled Trial. Gerontologist 2017; 57:707-18.

^193^Yeung WK, Tam WSW, Wong TW. Clustered randomized controlled trial of a hand hygiene intervention involving pocket-sized containers of alcohol-based hand rub for the control of infections in long-term care facilities. Infect Control Hosp Epidemiol 2011;32:67-76.

^194^Yokoi K, Yoshimasu K, Takemura S, Fukumoto J, Kurasawa S, Miyashita K. Short stick exercises for fall prevention among older adults: a cluster randomized trial. Disabil Rehabil 2015;37:1268-76.

^195^Zwijsen SA, Smalbrugge M, Eefsting JA, Twisk JWR, Gerritsen DL, Pot AM, et al. Coming to grips with challenging behavior: a cluster randomized controlled trial on the effects of a multidisciplinary care program for challenging behavior in dementia. J Am Med Dir Ass 2014;15: 531e1–e10.
